# Genome-Wide Association Study and Pathway Analysis for Heterophil/Lymphocyte (H/L) Ratio in Chicken

**DOI:** 10.3390/genes11091005

**Published:** 2020-08-27

**Authors:** Jie Wang, Bo Zhu, Jie Wen, Qinghe Li, Guiping Zhao

**Affiliations:** State Key Laboratory of Animal Nutrition, Institute of Animal Sciences, Chinese Academy of Agricultural Sciences, Beijing 100193, China; iwangjie0226@163.com (J.W.); bofenghouxizhuhou@163.com (B.Z.); wenjie@caas.cn (J.W.); liqinghe@caas.cn (Q.L.)

**Keywords:** H/L, GWAS, pathway, disease-resistant breeding

## Abstract

Disease control and prevention have been critical factors in the dramatic growth of the poultry industry. Disease resistance in chickens can be improved through genetic selection for immunocompetence. The heterophil/lymphocyte ratio (H/L) in the blood reflects the immune system status of chickens. Our objective was to conduct a genome-wide association study (GWAS) and pathway analysis to identify possible biological mechanisms involved in H/L traits. In this study, GWAS for H/L was performed in 1317 Cobb broilers to identify significant single-nucleotide polymorphisms (SNPs) associated with H/L. Eight SNPs (*p* < 1/8068) reached a significant level of association. The significant SNP on GGA 19 (chicken chromosome 19) was in the gene for complement C1q binding protein (*C1QBP*). The wild-type and mutant individuals showed significant differences in H/L at five identified SNPs (*p* < 0.05). According to the results of pathway analysis, nine associated pathways (*p* < 0.05) were identified. By combining GWAS with pathway analysis, we found that all SNPs after QC explained 12.4% of the phenotypic variation in H/L, and 52 SNPs associated with H/L explained as much as 9.7% of the phenotypic variation in H/L. Our findings contribute to understanding of the genetic regulation of H/L and provide theoretical support.

## 1. Introduction

With the continuous increase in intensive agriculture, the poultry industry has developed rapidly. Disease control and prevention have been critical factors in the dramatic growth of the poultry industry. The widespread distribution of poultry and high-intensity rearing has allowed infectious diseases to be spread and transmitted rapidly, resulting in considerable economic losses [1]. Vaccination is a common method of disease prevention and treatment, but it has raised concerns regarding expense and meat safety. Poultry has led the way among agricultural animal species in infectious disease control and, in particular, selection for genetic resistance. Disease resistance in chickens can be improved through genetic selection for immunocompetence. Heterophils are unique immune cells of birds and have the immune defense function of phagocytosis of pathogens in the innate immune system [2]. Heterophils play a crucial role in killing foreign pathogenic microorganisms when the specific immune system of the body is not mature. The ratio of heterophils to lymphocytes (H/L) is indicative of an interplay between immunity, physiology, and ecology. Studies have shown that the H/L is a characteristic indicator of the environmental adaptation and different degrees of genetic evolution of birds. The pathogen exposure hypothesis suggests that H/L decreases as the bird’s reproductive latitude decreases and is related to the degree of exposure to pathogens or parasites in different geographic environments [3]. The H/L in the peripheral blood of chickens has been widely accepted as a reliable and accurate physiological indicator of the chicken stress response [4]. With high or low temperatures, bacterial infection, and other stress reactions, the number of heterophils increases [5,6], and thus H/L can be used as a blood index reflecting the disease resistance of the body [4,7,8]. Two groups of chicken were selected as resistant (low H/L) and sensitive (high H/L) on the basis of H/L. Many aspects of immune response were compared after ST infection, namely H/L, antibody titer, cellular immunity, phagocytic activity, cortisol concentration, bursa, and body weight. The low H/L group exceeded the high H/L group in all the studied variables of the immune response [9].

Genome-wide association study (GWAS) can provide preliminary results in genetic research on complex traits in animals. Many single-nucleotide polymorphism (SNP) loci and qualitative trait loci (QTLs) with strong statistical significance have been discovered [10,11,12,13]. GWAS has become a powerful tool for studying important economic traits of livestock and poultry and has promoted research on marker-assisted breeding. However, GWAS cannot be used to identify the causative variation directly associated with the traits. The correlation indicates that a linked region is related to a specific trait. However, pinpointing the causative variation is more difficult. To reveal the mechanism through which a mutation site affects a phenotype and to perform subsequent functional research, GWAS joint analysis on multiple genomic levels can be used and biological pathway analysis can be applied to detect the superposition of multiple minor genes by examining genes involved in the same biological pathway, thus enabling deeper mining of GWAS data [14,15,16,17]. With the development of genome sequencing technology and the continuous improvement of statistical methods, GWAS is expected to become more efficiently applied to gene identification for important traits in livestock and poultry and play an increasingly important role in animal breeding.

Our goal was to identify the candidate genes associated with H/L disease resistance traits, with the goal of mitigating damage, and to conduct a pathway analysis to complement previously obtained GWAS results for phenotypes associated with H/L.

## 2. Materials and Methods

### 2.1. Ethics Statement

The work was approved by the Animal Management Committee of the Institute of Animal Sciences, Chinese Academy of Agricultural Sciences (IAS-CAAS, Beijing, China). Ethical approval regarding animal survival was granted by the animal ethics committee of IAS-CAAS (approval number: IASCAAS-AE20140615).

### 2.2. Phenotypes and Breeding Value Estimation

In 42-day-old chickens, 10 µL of blood was freshly obtained from the wing. Slides were air-dried and dyed with May–Grunwald–Giemsa stain, and the H/Ls were calculated. The basic data have been used in recent GWAS analysis, and details regarding the genotyping and phenotyping have been reported [18]. In this study, a conventional pedigree-based best linear unbiased prediction model (BLUP) was used to predict breeding values for H/L of the genotyped samples.

The pedigree-based BLUP model [19] is given as follows:

y=Xb+Za+e

where *y* is the vector of the phenotypic records of the trait (H/L), b is the vector of the fixed effect (Batch and Sex), X is the incidence matrix linking b to y, a is a vector of additive breeding values to be estimated, Z is the incidence matrices linking a to y, and e is the vector of residuals. We assumed that $\mathrm{var}\left(a\right)=A\sigma_{a}^{2}$, where A is the pedigree-based genetic relationship matrix. The prediction of breeding values (ebv) was performed with the Asreml package [20]. The ebv calculated in this part was used for the next GWAS analysis.

### 2.3. Genotyping and Quality Control

Briefly, 1317 birds were genotyped with 55K Affymetrix Axiom Chicken Genotyping Array [21] with the Affymetrix GeneTitan system according to the procedure described in the Axiom 2.0 Target Prep 384 Samples Protocol (Affymetrix; Santa Clara, CA, USA) (https://www.thermofisher.com/). Stringent quality control (QC) procedures were performed for the genotype data by using PLINK [22] version 1.09 (http://zzz.bwh.harvard.edu/plink/). After QC (call rate >95%, minor allele frequency >0.05, and extreme deviation from Hardy–Weinberg proportions (P > 1E-6)), 1212 animals and 36,750 SNPs, located on 28 autosomes and in the Z chromosome, were retained. Slight differences in the number of individuals and SNPs across the recent GWAS analyses are attributed to phenotypic editing.

### 2.4. Single SNP-Based Association Analysis

The population structure was assessed by principal component analysis (PCA) using GAPIT packages [19]. The basic MLM model was used for association analysis in GAPIT [23]. The *p*-value was corrected with a strict Bonferroni adjustment based on LD pruning [24]. The sums of the independent LD blocks plus singleton markers were used to define the number of independent statistical comparisons [25]. Finally, 8068 independent SNPs were used to determine the *p*-value thresholds, including 5% genome-wide significance (6.80 × 10^−6^, 0.05/8068), and suggestive association (1.24 × 10^−4^, 1/8068). The quantile–quantile (Q–Q) plot and Manhattan plots of GWAS for H/L were produced with the “qqman” packages [26] in R. The most significant SNP genotypes were added to univariate models to analyze the phenotypic differences among individuals with different SNP genotypes. Genes in a specific genomic region were detected by using Variant Effect Predictor (VEP) based on the GRCg6a assembly supported by Ensembl (http://asia.ensembl.org/Gallus_gallus/Info/Index).

### 2.5. Pathway Analysis

**Figure 1:**
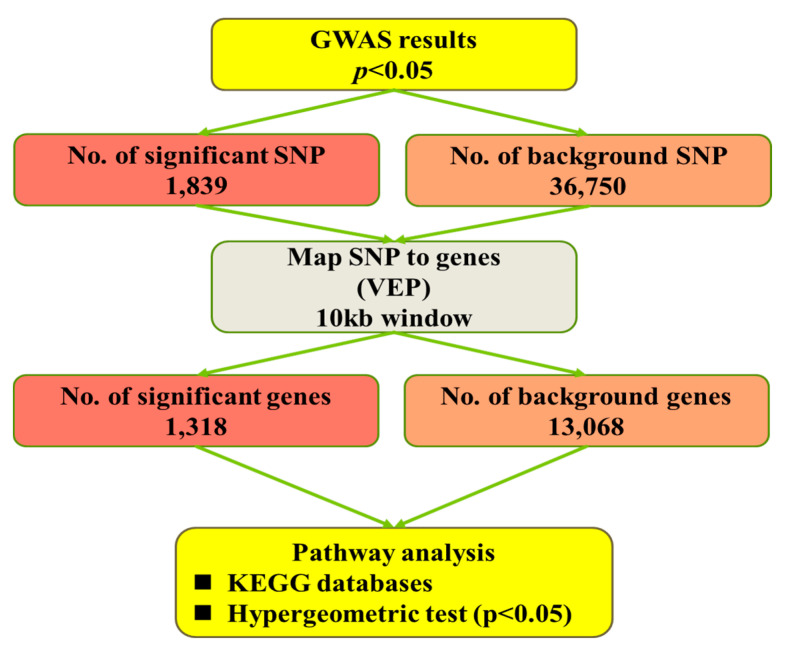
Flowchart for pathway-based analysis. GWAS = genome-wide association study; KEGG = Kyoto Encyclopedia of Genes and Genomes; VEP: Variant Effect Predictor; SNP: Single-Nucleotide Polymorphism.

The pathway-based analysis workflow is represented in Figure 1. In brief, for each trait, nominal *p*-values < 0.05 from the GWAS analyses were used to identify significant SNPs. With VEP software (http://asia.ensembl.org/Tools/VEP), SNPs were assigned to genes if they were within the genomic sequence of a gene or within a flanking region of 5 kb up- or downstream of a gene, to include SNPs located in regulatory regions. The Ensembl GRCg6a database was used as a reference (http://asia.ensembl.org/Gallus_gallus/Info/Index). The background SNPs represent all SNPs tested in the GWAS analyses, whereas the background genes were the genes associated with those SNPs. For assignment of the genes to functional categories, the Kyoto Encyclopedia of Genes and Genomes (KEGG) pathway [27] databases were used. Pathway enrichment analysis was performed with the OmicShare tools, a free online platform for data analysis (www.omicshare.com/tools). Significantly enriched pathways in significant genes compared with the background genes were defined with a hypergeometric test.

### 2.6. Proportion of Phenotypic Variance Explained (PVE) by SNPs

PVE estimation is equivalent to calculating the heritability of SNPs as $h_{snp}^{2}=\sigma_{g}^{2}/\sigma_{p}^{2}$. The GBLUP model was used to estimate the phenotypic variance explained by SNPs for all genotyped individuals [28]:

y=Xb+Zg+e

where the definitions of *y*, X, Z, and e are the same as those in the BLUP model; g is the vector of genomic breeding values to be estimated, following a normal distribution of N (0, G*σ_g_*^2^), in which *σ_g_*^2^ is the variance of additive genetic effect; and G is the marker-based genomic relationship matrix. Z is the incidence matrix linking g to *y*, and e is the vector of residuals; i.e., e is the vector of random errors, following a normal distribution of N (0, I*σ_e_*^2^), in which *σ_e_*^2^ is the residual variance. The estimation of variance components was performed with the Asreml package [20].

## 3. Results

### 3.1. Single SNP-Based Association Study

**Figure 2:**
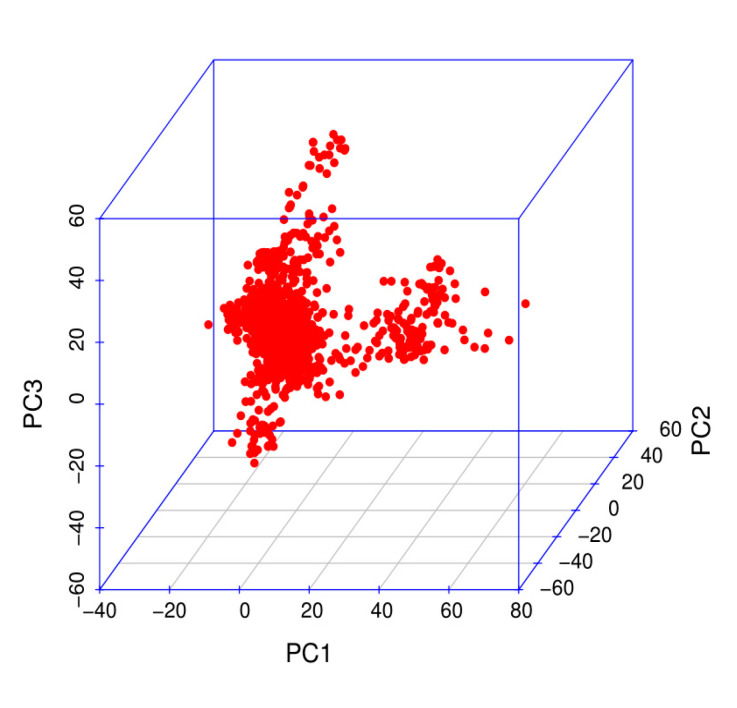
Population structure evaluated on the basis of the first three principal components.

In this study, 1317 birds were genotyped with Affymetrix Chicken 55K genotyping arrays. According to the QC criteria, 1212 animals and 36,750 SNPs were used in subsequent analyses. A total of 36,750 SNP markers were obtained with PCA by using the first three principal components (Figure 2). *p*-value correction was first performed with PLINKv1.9 software [19] for block analysis. Bonferroni correction was used to correct the *p*-values, and 8068 independent linkage disequilibrium (LD) blocks were obtained.

**Figure 3:**
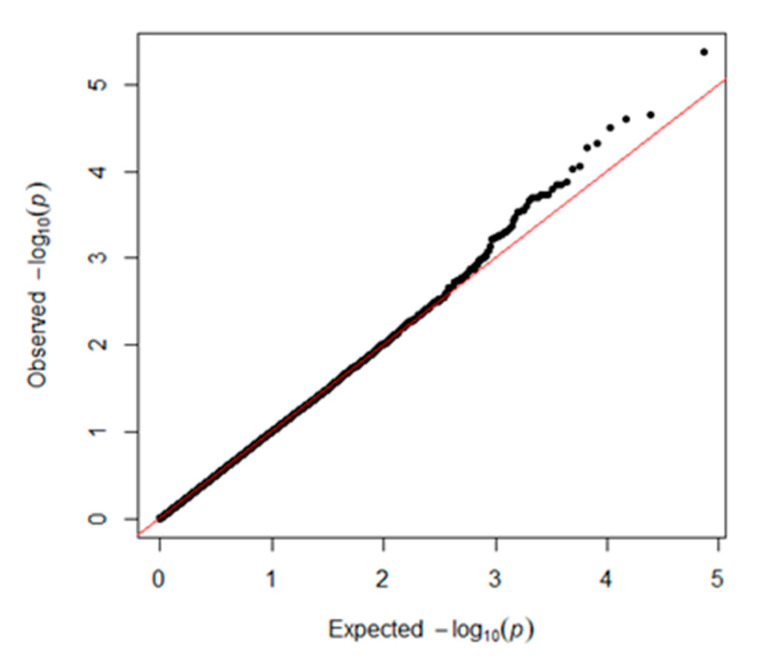
Quantile–quantile (Q–Q) plot of the GWAS results with GAPIT 3.0. The x-axis shows the expected *p*-values under the null hypothesis and the y-axis shows the observed *p*-values.

**Figure 4:**
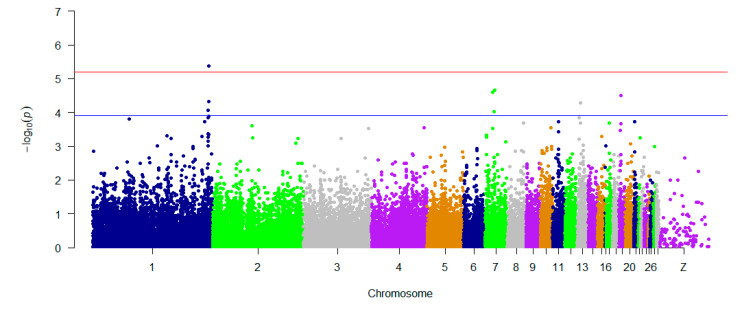
Manhattan plots showing association of all SNPs with the H/L trait, by using GAPIT 3.0. SNPs are plotted on the x-axis according to their positions on each chromosome, and their association with H/L is shown on the y-axis (as -log10 (*p*-value)). The blue dashed line indicates suggestive genome-wide significance (*p*-value = 1.24 × 10^−4^), and the red line shows genome-wide 5% significance with a *p*-value threshold of 6.80 × 10^−6^.

GAPIT was used for traditional SNP-based association analysis. In total, one associated SNP (5% genome-wide significance (6.80 × 10^−6^, 0.05/8068)) on GGA1 (chicken chromosome 1) and seven SNPs with a suggestive association (1.24 × 10^−4^, 1/8068) on GGAs 1, 7, 13, and 19 were detected. The detailed information on the SNPs reaching 5% genome-wide significance and the seven SNPs with a suggestive association is shown in Table 1. One of the genes found in the 100 kb region on chromosome 1 (ENSGALG00000041225) is a novel gene. *HNRNPA3* (heterogeneous nuclear ribonucleoprotein A3) is a protein-coding gene that plays a role in the cytoplasmic trafficking of RNA. *PLEKHM3* (pleckstrin homology domain containing M3) may act as a scaffold protein for AKT1 during muscle differentiation. *C1QBP* (complement C1q binding protein) is believed to be a multifunctional and multicompartmental protein involved in inflammation and infection processes, and its signaling has been implicated in inhibition of the innate immune response via the PI3K-AKT/PKB pathway. *TENM2* (teneurin transmembrane protein 2) is a protein-coding gene that mediates axon guidance and heterophilic cell–cell adhesion. *ZNF385B* (zinc finger protein 385B) may play a role in p53/TP53-mediated apoptosis. The Q–Q plot is presented in Figure 3. The global view of *p*-values (in terms of -log10 (*p*-value)) for all SNPs is represented by a Manhattan plot in Figure 4. The raw results of all SNPs are described in Table S1.

To effectively identify SNPs, we performed additional analyses, and the SNPs significantly associated with H/L and H/L ebv were fitted into the model to examine these associations. We suggest that the five identified loci provide the most reliable signal. The substitution of variant wild-type for mutant SNPs led to a significant decrease in H/L and H/L ebv. The results for all SNPs are described in Figure S1.

### 3.2. Pathway Analysis

**Figure 5:**
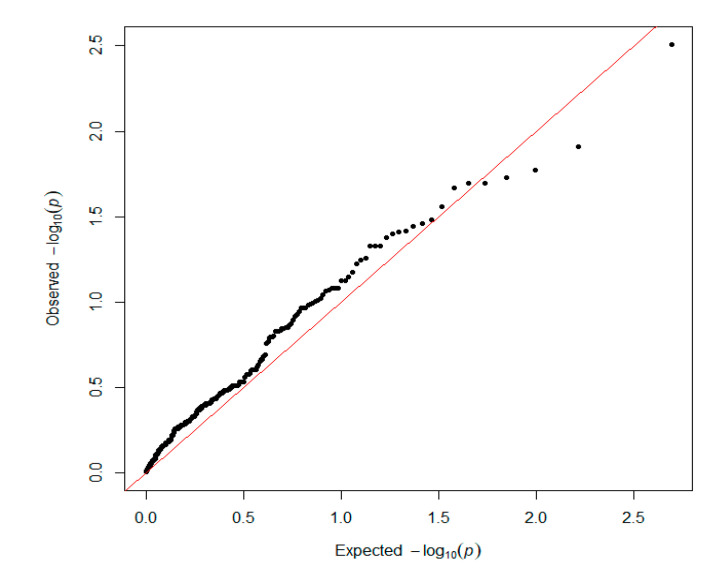
Q–Q plot for the SNPs used in the pathway-based analysis. The x-axis shows the expected *p*-values under the null hypothesis and the y-axis shows the observed *p*-values.

The same genotyping dataset was used in this analysis. On the basis of the QC and SNP selection criteria (Section 2), 335 pathways were selected from the KEGG dataset, covering 13,068 genes. In brief, nominal *p*-values < 0.05 from the GWAS analyses were used to identify significant SNPs. In total, 1839 SNPs (out of the 36,750 tested) were located in annotated genes or in the 10 kb window (upstream or downstream of a gene). The H/L trait was analyzed, and we identified nine associated pathways (*p* < 0.05): small cell lung cancer, proteoglycans in cancer, calcium signaling pathway, microRNAs in cancer, sulfur relay system, pathways in cancer, gastric acid secretion, purine metabolism, and salivary secretion. The Q–Q plot is shown in Figure 5. The detailed information for each pathway is provided in Additional file: Tables S2 and S3. Furthermore, we prepared plots of the number of genes for each pathway class to investigate the gene function. The results of gene classification confirmed the accuracy of the GWAS analysis (Figure S2).

### 3.3. Estimation of Phenotypic Variance Explained by SNPs from Various Datasets

**Figure 6:**
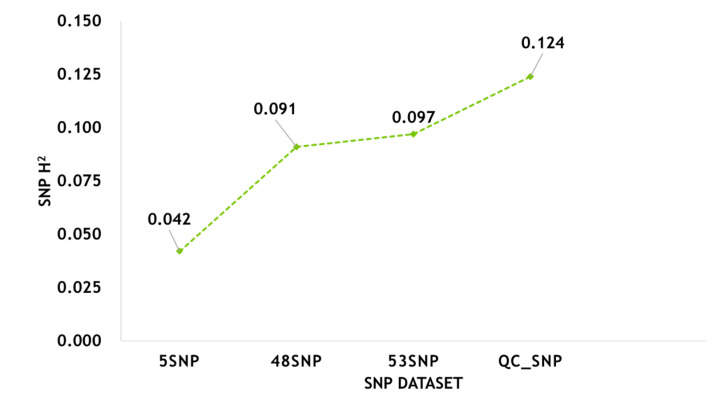
The contribution of SNPs from different datasets to the variation in H/L.

To determine the contribution of SNPs to the phenotypic variation in H/L, we estimated the phenotypic variance explained by SNPs from various datasets (Table S4, Figure 6). We analyzed all SNPs after QC (QC_SNP, 36,750 SNPs) and found that the phenotypic variation that they were able to explain was 12.4%. The phenotypic variation explained by GWAS-based loci (5 SNPs) and pathway-based loci (48 SNPs) was 4.2% and 9.1%, respectively. The phenotypic variation in H/L that could be explained by SNPs (53 SNPs, GWAS- and pathway-based) identified jointly by the two analyses was as high as 9.7%.

## 4. Discussion

### 4.1. Key Genes Associated with the H/L

Heterophils are a special type of white blood cells in poultry, which are responsible for immune defense and immune regulation in the body’s innate immune system. Similarly to mammalian neutrophils, heterophils mainly kill pathogens through trapping, phagocytosis, oxidative bursts, cytokine production, and other processes, thus protecting poultry from invasion [2]. The process through which phagocytic leukocytes produce reactive oxygen species (ROS) under external stimuli (such as microorganisms and microorganism-related molecules) is called oxidation or the respiratory burst. When the body is infected by pathogenic microorganisms and produces an acute inflammatory reaction, heterophils migrate to the inflammatory site. *C1QBP* mediates many biological responses in the immune system, including inflammation, infection, and immune regulation [29]. Mechanistically, *C1QBP* is a direct target gene of *ZNF32*; it inactivates the p38 mitogen-activated protein kinase (MAPK) pathway, thereby exerting the protective effects of ZNF32 against oxidative-stress-induced apoptosis. *C1QBP* is essential for sustaining mitochondrial membrane potential, and it enhances cellular resistance to oxidative stress [30]. *C1QBP* promotes the release of cytokines such as TNF-α, IL-1β, IL-6, IL-8, and MCP-1 from mononuclear cells [31,32]. The C1QBP-enhanced phagocytic process occurs through the activity of phosphoinositide 3-kinase (PI3K) [33,34]. *C1QBP* also plays an essential role in CSF-1 induced migration of macrophages [35].

ENSGALG00000041225 is a novel gene, encoding SH3 domain-containing protein. The SRC homology 3 domain (or SH3 domain) is a small protein domain of approximately 60 amino acid residues. This domain has been identified in several protein families such as PI3K, Ras GTPase-activating protein, CDC24, and CDC25. SH3 domains are found in proteins involved in signaling pathways regulating the cytoskeleton, such as the Ras protein, the Src kinase, and many others. Because PI3K and Src kinase are key enzymes in heterophils, this gene may be associated with heterophil function [36]. This novel gene identified may be related to the function of PI3K. *C1QBP* exerts immune regulation function through PI3K. These two genes may be located in the same regulatory network and play a role together.

### 4.2. Signaling Pathways Associated with the H/L

The regulation of the H/L may be a result of complex interactions among multiple pathways. According to the pathway analysis, several well-known pathways associated with immune response were found to be enriched (e.g., small cell lung cancer, proteoglycans in cancer, microRNAs in cancer, and pathways in cancer). In addition, other signaling pathways (calcium signaling pathway, sulfur relay system, purine metabolism, and salivary secretion) were enriched significantly (*p* < 0.05).

The sulfur relay system pathway, regulated by *MPST* and *URM1*, was significantly associated with H/L. According to previous research, *MPST* catalyzes the transfer of a sulfur ion from 3-mercaptopyruvate to cyanide or other thiol compounds. *MPST* is required for thiosulfate biosynthesis, and it contributes to the catabolism of cysteine. It is also an important producer of hydrogen sulfide (H_2_S) [37,38]. Treatment with H_2_S increases the proliferation and viability of LPS-treated L6 cells. H_2_S decreases ROS-induced apoptosis through the MAPK signaling pathway in LPS-treated L6 cells. H_2_S ameliorates LPS-induced inflammation through the ROS/MAPK and TLR4/NF-κB signaling pathways [39]. Both endogenous and exogenous H_2_S inhibit cell proliferation and cytokine release, in addition to inhibiting ERK-1/2 and MAPKs, thus resulting in a clear anti-inflammatory response [40]. H_2_S influences multiple biological functions of cells through inhibiting the PI3K/Akt/mTOR signaling pathway and consequently inhibiting cell migration, proliferation, and cell division [41].

### 4.3. Contribution of SNPs to the Variation in H/L

The dissection of the H/L effect was based on the theory of quantitative polygenic micro-effects. H/L is a complex trait affected by multiple genetic loci, but SNPs significantly associated with H/L, according to GWAS analysis results, can usually explain only part of phenotypic variation. This partial explanation is primarily because of interactions among genes or between genes and the environment, and the phenotypic variation that can be explained by SNPs is usually much lower than that of heritability in a narrow sense (i.e., the ratio of additive genetic variance to phenotypic variance). In contrast, non-additive effects do not contribute to narrow-sense heritability, mainly because some causative variation sites affecting phenotypes do not reach the threshold of significance, or the causative variation is not in LD with SNPs that have been genotyped [42]. Another reason is that GWAS considers SNPs above a significance threshold, but a lack of SNPs that reach the threshold does not mean that there is no additive effect. We found that all SNPs after QC explained 12.4% of the phenotypic variation in H/L, and the phenotypic variation explained by phenotypic SNPs was between 4.2% and 9.7%. In the H/L GWAS, combined with the pathway correlation analysis, 53 SNPs associated with H/L explained as much as 9.7% of the phenotypic variation in H/L, a value close to the phenotypic variation in H/L that all SNPs were able to explain. These results also precisely indicate that SNPs significantly associated with H/L play a major role in the variation in H/L in all SNPs. Simultaneously, correlation analysis based on pathways can meaningfully supplement the results of GWAS analysis.

## 5. Conclusions

This study comprised a GWAS and pathway analysis to identify genes associated with chicken H/L traits, thus revealing genes associated with H/L. The gene *C1QBP*, identified as associated with H/L estimated breeding value, may be an important candidate gene involved in the regulation of H/L. We also provide five SNPs that could be used as genetic markers in breeding programs. To our knowledge, this is the first GWAS and pathway analysis related to H/L ebv. In GWAS and animal breeding, studies have mainly focused on simple associations of SNPs with traits. The pathway analysis provided new findings relative to previous analysis, and confirmed that complex traits are affected by the cumulative effects of several genes clustered in specific biological pathways. The pathways found to be associated with traits may be useful in further studies involving gene mapping and development of breeding programs using SNP markers. Our findings provide new insights into the genetic mechanism underlying H/L.

## Figures and Tables

**Table 1:** Single-nucleotide polymorphisms (SNPs) with genome-wide significance for the heterophil/lymphocyte ratio (H/L).

Traits	SNP	CHR	POS	*p*-Value	Nearest Gene
H/L ebv	AX_172586886	1	189449719	4.23 × 10^−6^	ENSGALG00000041225 (U52kb)
H/L ebv	AX_76986680	7	15894092	2.20 × 10^−5^	*HNRNPA3* (within)
H/L ebv	AX_172565168	7	12098710	2.53 × 10^−5^	*PLEKHM3* (within)
H/L ebv	AX_75926692	19	3405344	3.18 × 10^−5^	*C1QBP* (within)
H/L ebv	AX_75383308	1	189419246	4.83 × 10^−5^	ENSGALG00000041225 (U21kb)
H/L ebv	AX_172582339	13	5407991	5.28 × 10^−5^	*TENM2* (D102Kb)
H/L ebv	AX_75383086	1	189344794	8.56 × 10^−5^	ENSGALG00000041225 (within)
H/L ebv	AX_76984294	7	14956470	9.37 × 10^−5^	*ZNF385B* (U24Kb)

## Supplementary Materials

The following are available online at https://www.mdpi.com/2073-4425/11/9/1005/s1: Figure S1: The relationship between separated SNPs with pleiotropic effect and H/L. Figure S2: Bar plot of KEGG pathway annotation for all significant genes. Table S1: Raw Results of All the SNPs. Table S2: Pathway Enrichment. Table S3: Pathway Detail. Table S4: Estimation of phenotypic variance.
