# Supplementary material for: Genome-Wide Association Study and Pathway Analysis for Heterophil/Lymphocyte (H/L) Ratio in Chicken
Source: Genes (Basel). 2020 Aug 27;11(9):1005. doi: 10.3390/genes11091005 (PMC7563235; doi:10.3390/genes11091005)
Supplement: Supplementary file 1 [file genes-11-01005-s001.zip › supplementary/Figures.docx]

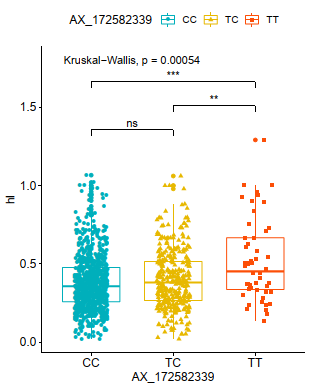

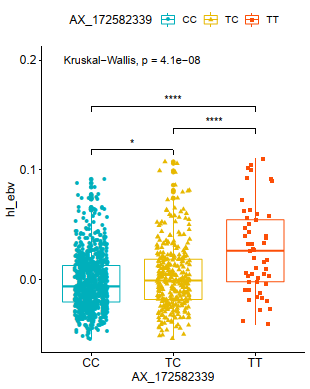

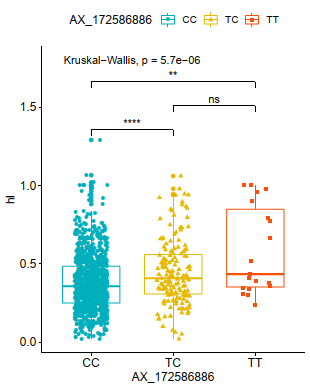

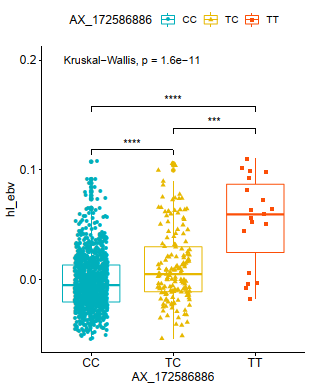

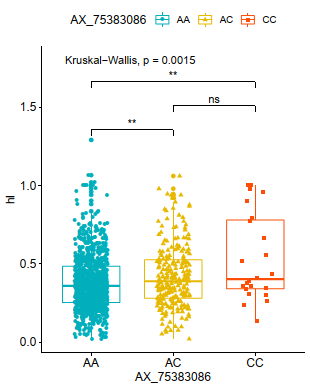

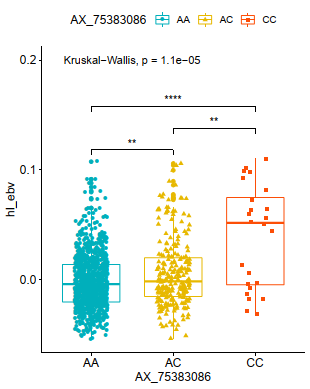

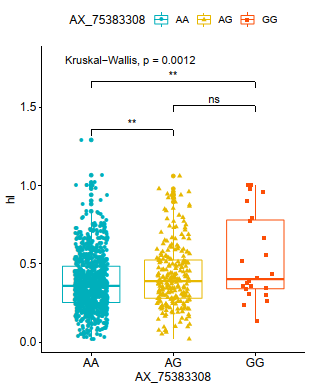

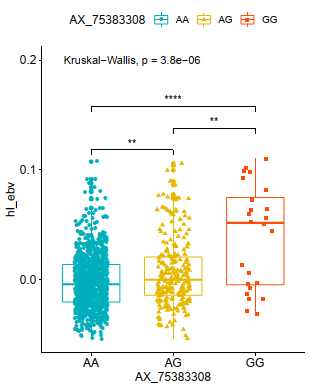

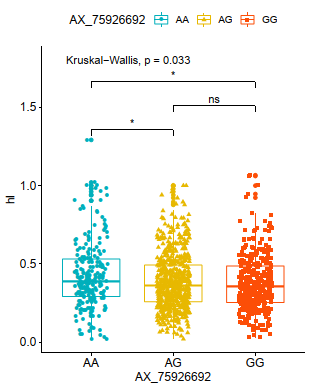

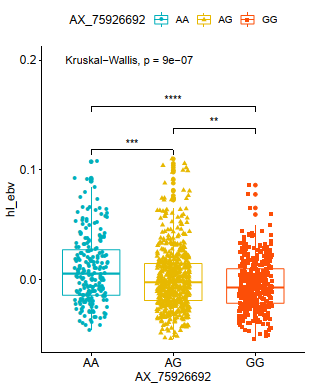


a

b

c

d

e

Figure S1. The relationship between separated SNPs with pleiotropic efffect and H/L. (**a**) Genotype effect plot of SNP AX_172582339 to indicate the significance among three types (n = 821, n = 330, and n = 52 for CC, TC, and TT, respectively). (**b**) Genotype effect plot of SNP AX_172586886 to indicate the significance among three types (n = 1025, n = 159, and n = 19 for CC, TC, and TT, respectively). (**c**) Genotype effect plot of SNP AX_75383086 to indicate the significance among three types (n = 936, n = 243, and n = 24 for AA, AC, and CC, respectively). (**d**) Genotype effect plot of SNP AX_75383308 to indicate the significance among three types (n = 939, n = 240, and n = 24 for AA, AG, and GG, respectively). (**e**) Genotype effect plot of SNP AX_75926692 to indicate the significance among three types (n = 219, n = 596, and n = 388 for AA, AG, and GG, respectively).


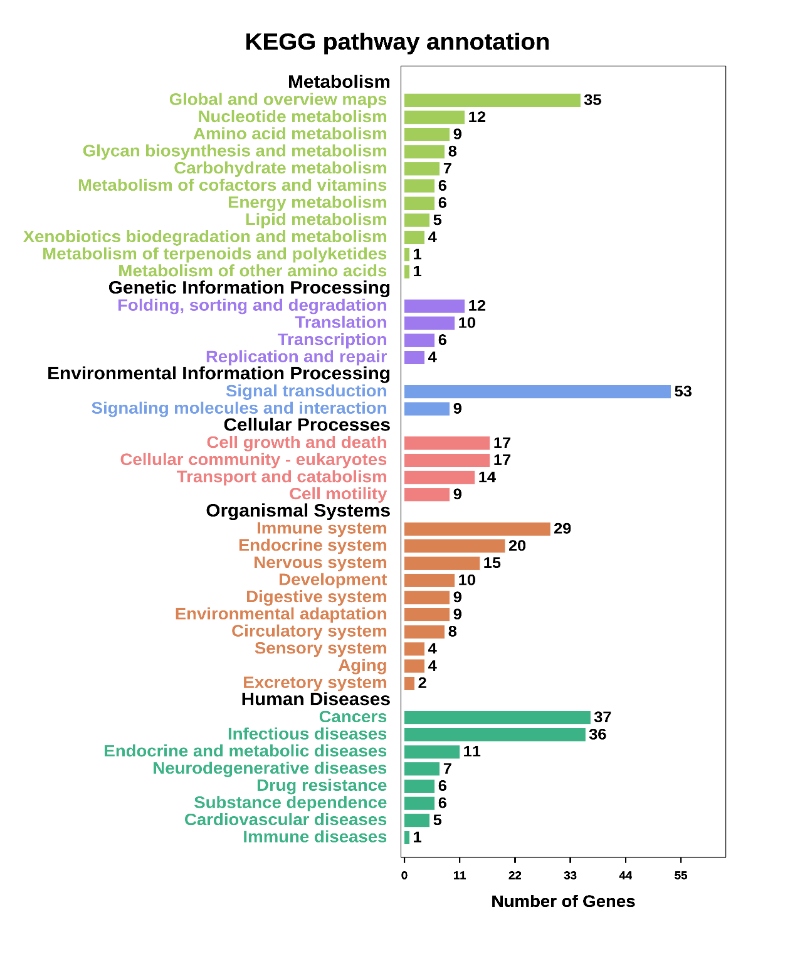


Figure S2 Bar plot of KEGG pathway annotation for all significant genes
